# Prediction Model for Gastric Cancer Incidence in Korean Population

**DOI:** 10.1371/journal.pone.0132613

**Published:** 2015-07-17

**Authors:** Bang Wool Eom, Jungnam Joo, Sohee Kim, Aesun Shin, Hye-Ryung Yang, Junghyun Park, Il Ju Choi, Young-Woo Kim, Jeongseon Kim, Byung-Ho Nam

**Affiliations:** 1 Gastric Cancer Branch, Research Institute & Hospital, National Cancer Center, Goyang-si, Republic of Korea; 2 Biometric Research Branch, Division of Cancer Epidemiology and Prevention, Research Institute & Hospital, National Cancer Center, Goyang-si, Republic of Korea; 3 Molecular Epidemiology Branch, Division of Cancer Epidemiology and Prevention, Research Institute & Hospital, National Cancer Center, Goyang-si, Republic of Korea; National Cancer Center, JAPAN

## Abstract

**Background:**

Predicting high risk groups for gastric cancer and motivating these groups to receive regular checkups is required for the early detection of gastric cancer. The aim of this study is was to develop a prediction model for gastric cancer incidence based on a large population-based cohort in Korea.

**Method:**

Based on the National Health Insurance Corporation data, we analyzed 10 major risk factors for gastric cancer. The Cox proportional hazards model was used to develop gender specific prediction models for gastric cancer development, and the performance of the developed model in terms of discrimination and calibration was also validated using an independent cohort. Discrimination ability was evaluated using Harrell’s C-statistics, and the calibration was evaluated using a calibration plot and slope.

**Results:**

During a median of 11.4 years of follow-up, 19,465 (1.4%) and 5,579 (0.7%) newly developed gastric cancer cases were observed among 1,372,424 men and 804,077 women, respectively. The prediction models included age, BMI, family history, meal regularity, salt preference, alcohol consumption, smoking and physical activity for men, and age, BMI, family history, salt preference, alcohol consumption, and smoking for women. This prediction model showed good accuracy and predictability in both the developing and validation cohorts (C-statistics: 0.764 for men, 0.706 for women).

**Conclusions:**

In this study, a prediction model for gastric cancer incidence was developed that displayed a good performance.

## Introduction

Gastric cancer is the fourth most common cancer in the world, and approximately 1 million new cases are diagnosed annually worldwide [1]. Although the incidence has decreased substantially in most parts of the world, gastric cancer remains the most common cancer and the third most common cause of death from cancer in Korea [2,3].

The prognosis of patients with gastric cancer is highly different according to pathological stage. The 5-year survival rate of patients with stage IA gastric cancer is 95.1–98.9% in Korea; however, this Fig declines to 26.1–32.2% in patients with stage IIIC [4–6]. For the patients who undergo palliative chemotherapy for stage IV, an overall survival of approximately 1 year is expected worldwide [7,8]. This great difference of survival according to the stage suggests that early detection before tumor progression is important for a good prognosis. For early detection, regular screening is essential, and regular screening was reported to be associated with a lower mortality from gastric cancer in previous population-based cohort studies [9–11].

In Korea, the national gastric cancer screening program has been running since 1999 as a part of the National Cancer Screening Program [12]. The target population of the National Cancer Screening Program was less than 50% of National Health Insurance beneficiaries in 2005 and was extended to the entirely of the National Health Insurance beneficiaries in 2010. Therefore, all beneficiaries older than 40 were advised to undergo a gastroscopy or upper gastrointestinal series examinations every 2 years.

The screening rate was 34.4% in 2004 and increased to 64.6% in 2011. Nevertheless, a significant proportion of eligible patients still do not undergo gastric cancer screening. Public indifference to mass screening and the unawareness of the risk factors for developing gastric cancer might be related to the low screening rate. Therefore, the identification of high risk populations and the notification of such populations may have a significant effect on improving the survival rate.

A risk prediction model is a simple and effective method used to evaluated individualized risk by quantifying cancer risk. However, few studies have established risk prediction models for gastric cancer incidence using epidemiological risk factors [13]. In this study, we have conducted a systematic investigation of the potential risk factors of gastric cancer using a large population-based cohort in Korea, with the aim of developing a risk prediction model for gastric cancer incidence.

## Materials and Methods

### Study population

The study cohort consisted of Korean government employees, teachers, company employees and their dependents who underwent a biennial medical examination provided by the National Health Insurance Corporation (NHIC) between the years 1996 and 1997. After excluding the recipients who were under 30 or over 80 and who had a previous cancer history or who were diagnosed as with gastric cancer between the years 1996 and 1997, we identified 2,291,132 individuals (1,436,958 men and 854,174 women) with data on baseline characteristics. Ten risk factors were considered for modeling including age, body mass index (BMI), family history of any type of cancer, meal regularity, salt preference, frequency of meat consumption, dietary preference, alcohol consumption, smoking and physical activity. However, the NHIC data have a huge portion of missing data because most life style information was obtained by self-report questionnaires. After the recipients who had missing data on any one of the risk factors were excluded, only 823,741 (57.3%) men and 369,554 (43.3%) women remained.

The proportion of excluded recipients because of missing data was considerably high; thus, we complemented the data using the imputation method. We were able to do this because the NHIC examination was provided every two years, and we were able to retrieve some information from the NHIC examination data performed in the years other than 1996 and 1997. When a participant received multiple examinations, the nearest time point was used to impute the missing values. Finally, 1,372,424 (95.5%) men and 804,077 (94.1%) women were available for model development after imputation. The difference in prediction models developed based on the complete data and imputed data with the nearest observations was minor (S1 File); therefore, the model development and validation were based on the imputed, larger data set.

To evaluate the performance of the developed model, an independent population who underwent the National Health Insurance Corporation medical evaluation between the years 1998 and 1999 was used as a validation cohort. Among all eligible recipients, we excluded recipients who were included in the development model in addition to recipients who met same exclusion criteria. Similar missing data imputation was applied, and finally a total of 484,335 men (4.3% missing) and 466,013 women (3.5% missing) were included in the validation cohort.

This study was approved by the Institutional Review Board of the National Cancer Center, Korea (IRB no. NCCNCS 09–305).

### Data collection and risk factor assessment

During the health examination, the weight, height and blood pressure of each participant were measured as part of the routine physical examination. Additionally, the participants completed a questionnaire about family history of any type of cancer, previous disease history, dietary habits, alcohol consumption, smoking and physical activity. Each question had simple choices because it was self-recorded and the categories of diet habits and physical activity were subjective ones such as ‘Regular’, ‘Intermediate’, and’ Irregular’ for meal regularity, and ‘Not salty’, ‘Intermediate’, and ‘Salty’ for salt preference. Based on these simple questionnaires, the risk factors for gastric cancer development were analyzed.

### Cancer ascertainment and identification of death

Data for gastric cancer incidence were obtained from the Korean Central Cancer Registry database through December 31, 2007. Based on the International Classification of Disease, 10th edition, C16 was used for the incidence of gastric cancer. Deaths and causes of death were identified from the death records of the National Statistical Office, which is a nationwide registration of deaths, and the National Health Insurance Corporation.

### Statistical analysis

A Cox proportional hazards regression model was used to estimate the relative risks (and corresponding 95% confidence intervals (CIs)) of gastric cancer incidence for each of the potential risk factors. The proportionality in hazards was examined via log-log survival plots. We noticed that the demographic characteristics and environmental exposures were different between men and women, and both crude and age (at baseline) adjusted analyses were performed separately for men and women.

The potential risk factors considered in the analysis were BMI (<18.5, 18.5–22.9, 23.0–24.9, and ≥25), family history of any type of cancer (yes or no), meal regularity (regular, intermediate, irregular), salt preference (not salty, intermediate, salty), frequency of meat consumption (≤1, 2–3, and ≥4 times per week), dietary preference (vegetables preferred, mixed, and meat preferred), alcohol consumption (none, i.e., 0 g; light, i.e., 1–14.9 g; moderate, i.e., 1.5.0–24.9 g; and heavy, i.e., ≥25 g of ethanol per day), smoking (never, former, current < 10, current 10–19, and current ≥ 20 cigarettes per day), and physical activity (none, light, moderate, and heavy). For women, because of the small number of incidences, several categories of the alcohol consumption and smoking variables were combined. For alcohol consumption, those with more than 15 g of ethanol were combined, and only two categories were used for smoking (never, smoker). Further descriptions of the rationale of the categorizations of these variables can be found elsewhere [14,15].

A backward variable selection method with a type I error criterion of 0.1 based on likelihood ratio tests was considered in the multivariable model. The probability of developing gastric cancer within t years (t = 8) for an individual with covariate values x = (x_1_,…, x_K_) for K risk factors can be estimated using the following equation:

$$1-{S_{0}(t)}^{\exp\left[f(x)\right]},\;where\;f(x)=\sum_{i}\beta_{i}x_{i}$$

Here, S_0_(t) is the mean survival probability at time t for an individual whose covariate values are all 0, and the β_i_ s are the estimated coefficients from the Cox proportional hazard model. Once β_i_ and S_0_(t) are obtained, the probability of developing gastric cancer for any set of covariate values can be estimated.

The developed models were validated in an independent cohort population by evaluating the performance of the models with respect to their discrimination ability using C-statistics, and the calibration ability was evaluated using a calibration plot and calibration slope [16–21].

Harrell’s C-statistics for survival data was considered in this study [18–20]. This value represents the probability that the predicted probability of developing gastric cancer is higher for those who actually develop gastric cancer in 8 years than for those who do not develop gastric cancer. Calibration is related to the accuracy of the prediction. To generate a calibration plot, the data were first divided into 10 disjointed subgroups according to the predicted probabilities of developing gastric cancer based on the developed model. The expected (the average predicted probabilities) and observed (the actual event rate measured by the Kaplan-Meier estimate) values were then plotted. Additionally, to obtain the calibration slope, the prognostic index (PI) from the Cox regression, which is the weighted linear combination of the variables selected for the prediction model, was obtained for individuals in the validation data set, and the regression coefficient on the PI was obtained. A PI close to 1 indicates good calibration, and a likelihood-ratio test that tests whether this slope is 1 is then performed [22].

All the analyses were performed using SAS (version 9.1.3; SAS Institute, Cary, NC) and STATA (version 13) software.

### Ethics statement

This study was performed with the approval of the institutional review boards of the National Cancer, Center, Korea (No. NCCNCS 09–305). The participants’ informed consent was waived by the institutional review boards because this study involved routinely collected medical data that were anonymously managed in all stages, including the stages of data cleaning and statistical analyses.

## Results

### Cancer incidence and baseline characteristics

The total number of person-years of follow-up was 14,815,612 for men and 8,471,357 for women for a median of 11.3 years of follow-up. The mean (SD) ages of the men and women were 45.1 (10.5) and 48.7 years (11.0), respectively. During follow-up, 19,465 (1.4%) and 5,579 (0.7%) cases of gastric cancer were observed among 1,372,424 men and 804,077 women, respectively, resulting in incidence rates of 131.38/100,000 and 65.89/100,000 person years for each sex. In the validation cohort, a total of 6,628 and 2,920 gastric cancer cases were observed out of 484,335 men and 466,013 women, respectively. The incidence rates in the validation cohort were 164.54/100,000 for men and 75.84/100,000 for women; these rates were higher than those observed in the model developing cohort.

### Risk factors

To evaluate the significant risk factors of gastric cancer incidence, a multivariable analysis was performed based on the variable selection criteria. Tables 1 and 2 show the incidences of gastric cancer and the estimated hazard ratio for each of the potential risk factors for men and women, respectively. For men, the significant risk factors of gastric cancer incidence were age, low weight, having a family member who had previously had any type of cancer, irregular meals, salt preference, alcohol consumption, and smoking. Among these risk factors, a clear trend of increased risk was observed for alcohol consumption and smoking (linear trend test *P* <0.0001 for both variables). Heavy drinkers (ethanol ≥ 25 g/day) had a more than 20.4% increased risk, and heavy smokers (1 pack currently) had a more than 43.1% increased risk of gastric cancer incidence. Additionally, those who had a family member with any type of cancer had a 30.2% increased risk, and irregular meal consumption and a preference of salty food also conferred an increased risk. Conversely, a BMI ≥23 kg/m^2^ and moderate to high physical activity were protective factors.

**Table 1:** Risk factor distributions between gastric cancer patients and gastric cancer-free patients (men), and age-adjusted univariable and multivariable model in the developing cohort.

	Frequency			Age-adjusted univariable model			Multivariable model	
	No. of participants at baseline (N = 1,372,424)	No. of event (N = 19,465)	HR	95% CI	*p*	HR	95% CI	*p*
Age (year)								
Mean (SD)	45.08 (10.47)							
(Age-Mean_age_)						1.111	1.108, 1.113	< .0001
(Age-Mean_age_)^2^						0.999	0.998, 0.999	< .0001
BMI (kg/m^2^)								
<18.5	33,444	709	1.094	1.013, 1.181	0.0227	1.135	1.051, 1.226	0.0013
18.5–22.9	560,155	8,374	1			1		
23.0–24.9	388,672	5,297	0.927	0.895, 0.959	< .0001	0.919	0.887, 0.951	< .0001
≥25	390,153	5,085	0.907	0.876, 0.939	< .0001	0.895	0.864, 0.927	< .0001
Family history of cancer								
No	1,176,051	16,360	1			1		
Yes	196,373	3,105	1.321	1.271, 1.373	< .0001	1.302	1.253, 1.353	< .0001
Meal regularity								
Regular	801,088	12,109	1			1		
Intermediate	453,692	5,888	1.096	1.062, 1.131	< .0001	1.031	0.998, 1.064	0.0643
Irregular	117,644	1,468	1.175	1.112, 1.240	< .0001	1.069	1.011, 1.130	0.0187
Salt preference								
Not salty	221,163	2,941	1			1		
Intermediate	859,662	11,852	1.080	1.037, 1.124	0.0002	1.026	0.985, 1.068	0.2180
Salty	291,599	4,672	1.238	1.183, 1.297	< .0001	1.087	1.037, 1.140	0.0006
Meal preferences								
Vegetables	281,894	4,166	1					
Mixed	975,850	13,665	0.992	0.958, 1.027	0.6500			
Meat	114,680	1,634	1.014	0.958, 1.074	0.6282			
Meat consumption frequency (per week)								
≤1 time	633,170	9,142	1					
2–3 times	663,355	8,832	0.981	0.952, 1.010	0.1935			
≥4 times	75,899	1,491	1.013	0.959, 1.071	0.6374			
Alcohol consumption (g/day)								
0	412,381	6,303	1			1		
1–14.9	392,217	4,937	1.064	1.025, 1.105	0.0012	1.022	0.984, 1.062	0.2596
15–24.9	239,738	3,047	1.160	1.110, 1.212	< .0001	1.081	1.034, 1.130	0.0006
25 or more	328,088	5,178	1.333	1.284, 1.383	< .0001	1.204	1.158, 1.251	< .0001
Smoking amount								
Never	409,331	5,284	1			1		
Ex-smoker	201,535	3,137	1.196	1.144, 1.250	< .0001	1.152	1.102, 1.204	< .0001
0.5 pack currently	128,102	2,010	1.261	1.197, 1.327	< .0001	1.238	1.175, 1.304	< .0001
0.5–1 pack currently	447,550	6,327	1.441	1.389, 1.496	< .0001	1.349	1.299, 1.402	< .0001
1 pack currently	185,906	2,707	1.583	1.511, 1.659	< .0001	1.431	1.364, 1.502	< .0001
Physical activity								
None	661,395	9,753	1			1		
Low	218,528	2,991	0.998	0.958, 1.040	0.9419	1.001	0.960, 1.043	0.9619
Moderate to High	492,501	6,721	0.945	0.916, 0.975	0.0004	0.950	0.920, 0.981	0.0015

HR, hazard ratio; CI, confidence interval; BMI, body mass index; SD, standard deviation.

**Table 2:** Risk factor distributions between gastric cancer patients and gastric cancer-free patients (women), and age-adjusted univariable and multivariable model in the developing cohort.

	Frequency		Age-adjusted univariable model			Multivariable model		
	No. of participants at baseline (N = 804,077)	No. of event (N = 5,579)	HR	95% CI	*p*	HR	95% CI	*p*
Age (/year)								
Mean (SD)	48.74 (11.01)					1.072	1.068, 1.076	< .0001
(Age-Mean_age_)						0.999	0.999, 1.000	< .0001
(Age-Mean_age_)^2^								
BMI (kg/m^2^)								
<18.5	31,585	224	1.138	0.991, 1.306	0.0660	1.160	1.010, 1.333	0.0352
18.5–22.9	347,036	2,121	1			1		
23.0–24.9	195,059	1,410	1.017	0.951, 1.088	0.6265	0.993	0.928, 1.063	0.8380
≥25	230,397	1,824	0.998	0.937, 1.063	0.9580	0.966	0.906, 1.030	0.2882
Family history of cancer								
No	680,994	4,652	1			1		
Yes	123,083	927	1.284	1.196, 1.378	< .0001	1.274	1.187, 1.368	< .0001
Meal regularity								
Regular	454,733	3,227	1					
Intermediate	253,836	1,716	1.007	0.949, 1.067	0.8282			
Irregular	95,508	636	1.055	0.968, 1.148	0.2218			
Salt preference								
Not salty	128,382	907	1			1		
Intermediate	543,585	3,612	1.012	0.941, 1.089	0.7399	1.005	0.934, 1.081	0.8882
Salty	132,110	1,060	1.119	1.024, 1.223	0.0128	1.090	0.997, 1.192	0.0588
Meal preferences								
Vegetables	277,071	2,149	1					
Mixed	491,334	3,190	0.976	0.924, 1.032	0.3963			
Meat	35,672	240	1.041	0.911, 1.189	0.5568			
Meat consumption frequency (per week)								
≤1 time	447,406	3,176	1					
2–3 times	298,283	1,974	0.981	0.928, 1.038	0.5098			
≥4 times	58,388	429	0.974	0.880, 1.077	0.6089			
Alcohol consumption (g/day)								
0	685,094	4,805	1			1		
1–14.9	101,031	607	0.991	0.911, 1.079	0.8411	0.972	0.892, 1.058	0.5113
15 or more	17,952	167	1.240	1.063, 1.446	0.0063	1.162	0.994, 1.359	0.0600
Smoking amount								
Never	767,663	5,110	1			1		
smoker	36,414	469	1.265	1.149, 1.393	< .0001	1.257	1.140, 1.387	< .0001
Physical activity								
None	599,503	4,172	1					
Yes	204,574	1,407	0.997	0.938, 1.059	0.9192			

HR, hazard ratio; CI, confidence interval; BMI, body mass index; SD, standard deviation.

For women, the significant risk factors were age, BMI, having a family member who had any type of cancer, and former smoking. Salt preference and alcohol consumption had were marginally significant (< 0.1; these variables were thus included in the model), and meal regularity and physical activity had no effect on gastric cancer incidence in women.

### Prediction model

Based on the multivariable analysis results, we developed gender specific prediction models as follows. (A for men, B for women).

#### A. Risk prediction model for men

Step 1: Form a prognostic index (PI) using the β-coefficient estimates

$$\text{PI}=0.1050*\left[\left({\text{Age-Mean}}_{\text{age}}\right)\;\text{-}\;0\right]–0.0014*[{\left({\text{Age-Mean}}_{\text{age}}\right)}^{2}\;\text{-}109.7040]$$

**Table:** 

+0.1269*(BMI—0.0244)	if BMI < 18.5
+0.0*(BMI)	if BMI 18.5–22.9
−0.0847*(BMI—0.2832)	if BMI 23.0–24.9
−0.1109*(BMI—0.2843)	if BMI ≥ 25.0
+0.0*(Family history of cancer)	if Family history of cancer, none
+0.2640* (Family history of cancer—0.1431)	if Family history of cancer, yes
+0.0* (Meal regularity)	if Meal regularity, Regular
+0.0301* (Salt preference—0.3306)	if Meal regularity, Intermediate
+0.0666* (Salt preference—0.0857)	if Meal regularity, Irregular
+0.0* (Salt preference)	if Salt preference, Not salty
+0.0255* (Salt preference—0.6264)	if Salt preference, Intermediate
+0.0836* (Salt preference—0.2125)	if Salt preference, salty
+ 0.0*(Alcohol consumption)	if Alcohol consumption, none
+ 0.0218* (Alcohol consumption—0.2858)	if Alcohol consumption, 1–14.9 g/day
+ 0.0778* (Alcohol consumption—0.1747)	if Alcohol consumption, 15–24.9 g/day
+ 0.1856* (Alcohol consumption—0.2391)	if Alcohol consumption, 25 more g/day
+ 0.0*(Smoking amount)	if none Smoker
+ 0.1414* (Smoking amount—0.1468)	if Past Smoker
+ 0.2132* (Smoking amount—0.0933)	if Current Smoker, <0.5 pack/day
+ 0.2997* (Smoking amount – 0.3261)	if Current Smoker, 0.5–0.99 pack/day
+ 0.3587* (Smoking amount – 0.1355)	if Current Smoker, ≥1 pack/day
+ 0.0*(PhA)	if Physical Activity, none
+0.0010*(PhA—0.1592)	if Physical Activity, light
−0.0513*(PhA—0.3589)	if Physical Activity, moderate to high

Step 2: Calculate the probability *P* = 1 –S(t|t = 8)^Exp(PI)^In which S(t|t = 8) is the survival probability estimate for the mean values of the risk factors in the model. Here, S(t|t = 8) = 0.9939406.

#### B. Risk prediction model for women

Step 1: Form a prognostic index (PI) using the β-coefficient estimates

$$\text{PI}=0.0692*\left[\left({\text{Age-Mean}}_{\text{age}}\right)\;\text{-}\;0\right]–0.006*\left[{\left({\text{Age-Mean}}_{\text{age}}\right)}^{2}\;\text{-}\;121.1973\right]$$

**Table:** 

+0.1487*(BMI—0.0393)	if BMI < 18.5
+0.0*(BMI)	if BMI 18.5–22.9
−0.0071*(BMI—0.2426)	if BMI 23.0–24.9
−0.0347*(BMI—0.2865)	if BMI ≥ 25.0
+0.0*(Family history of cancer)	if Family history of cancer, none
+0.2424*(Family history of cancer—0.1531)	if Family history of cancer, yes
+0.0*(Salt preference)	if Salt preference, Not salty
+0.0052*(Salt preference—0.6760)	if Salt preference, Intermediate
+0.0862*(Salt preference—0.1643)	if Salt preference, salty
+ 0.0*(Alcohol consumption)	if Alcohol consumption, none
−0.0286*(Alcohol consumption—0.1256)	if Alcohol consumption, 1–14.9 g/day
+ 0.1502*(Alcohol consumption—0.0223)	if Alcohol consumption, 15 more g/day
+ 0.0*(Smoking)	if none Smoker
+ 0.2291*(Smoking—0.0453)	if Smoker(Past, Current)

Step 2: Calculate the probability *P* = 1 –S(t|t = 8)^Exp(PI)^

In which S(t|t = 8) is the survival probability estimate for the mean values of the risk factors in the model. Here, S(t|t = 8) = 0.9961374.

**Fig 1:**
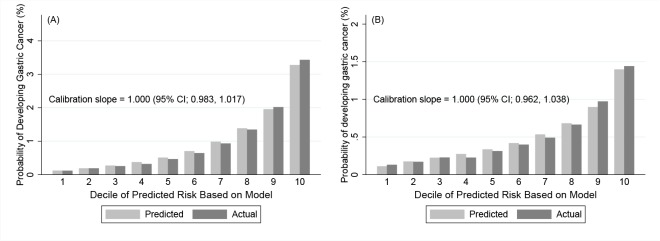
Calibration plots in the development cohort. (A) Calibration plots with calibration slopes for men (B) for women in the model developing cohort. The X-axis of the calibration plot corresponds to the deciles of predicted risk based on the model, and the Y-axis of the calibration plot corresponds to the probability of developing gastric cancer in 8 years (%).

The receiver operating characteristic curve analysis was performed to evaluate the discrimination ability of the developed model, and the C-statistics were 0.764 (95% CI, 0.760–0.768) for men and 0.706 (95% CI, 0.698–0.715) for women. The calibration ability was also evaluated by the calibration plot, and the predicted and actual probability of gastric cancer development appeared to be almost identical in each risk group (Fig 1(A) for men 1(B) for women).

### Model validation

In the validation cohort, the mean ages (SD) of men and women were 46.8 (12.8) and 51.1 years (12.1), respectively. The age-adjusted hazard ratios of the risk factors in the validation cohort are presented in Tables 3 and 4. Similar results were found for men, and only meal regularity had marginal significance. For women, a family history of cancer, salt preference, and vegetable preference were found to be significant risk factors, and a BMI ≥25 was a marginally protective factor. Unlike the model developing cohort, smoking was not a significant risk factor in the validation cohort. These results were possibly derived from the small event sizes and shorter follow-up period of the validation cohort compared to that of the model developing cohort.

**Fig 2:**
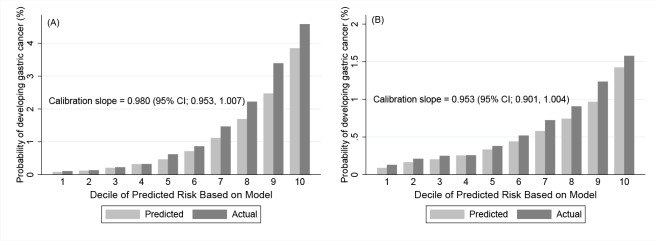
Calibration plots in the validation cohort. (A) Calibration plots with calibration slopes for men and (B) for women in the validation cohort. The X-axis of the calibration plot corresponds to the deciles of predicted risk based on the model, and the Y-axis of the calibration plot corresponds to the probability of developing gastric cancer in 8 years (%).

For the model validation, the 8 year survival rates of the patients in the validation cohort were estimated using the coefficients of the risk factors estimated from the original model developing cohort. Based on the estimated survival rates, the discrimination and calibration abilities of the model in the validation cohort were then obtained (Fig 2(A) for men 2(B) for women). The C statistics were 0.782 (95% CI, 0.777–0.787) and 0.705 (95% CI, 0.696–0.714) for men and women, respectively, and these discrimination abilities of the prediction model were as good as that in the model developing cohort. Fig 2(A) and 2(B) were calibration plots for each gender, and good calibration abilities were presented for gastric cancer development. The calibration slope, which is the regression coefficient of the PI using the validation data set, was 0.980 for men (*P* = 0.15) and 0.953 (*P* = 0.07) for women, which indicates good calibration.

**Table 3:** Risk factor distributions between gastric cancer patients and gastric cancer-free patients (men), and age-adjusted univariable model in the validation cohort.

	Frequency		Age-adjusted univariable model		
	No. of participants at baseline (N = 484,335)	No. of event (N = 6,628)	HR	95% CI	*p*
Age (/year)					
Mean (SD)	46.83 (12.80)				
(Age-Mean_age_)					
(Age-Mean_age_)^2^					
BMI (kg/m^2^)					
<18.5	15,748	402	1.174	1.058, 1.304	0.0026
18.5–22.9	193,460	3,021	1		
23.0–24.9	126,872	1,628	0.929	0.875, 0.987	0.0177
≥25	148,255	1,577	0.847	0.796, 0.901	< .0001
Family history of cancer					
No	424,308	5,853	1		
Yes	60,027	775	1.235	1.145, 1.332	< .0001
Meal regularity					
Regular	272,060	4,233	1		
Intermediate	165,126	1,921	1.051	0.995, 1.110	0.0743
Irregular	47,149	474	1.100	1.000, 1.211	0.0511
Salt preference					
Not salty	73,287	969	1		
Intermediate	293,652	3,845	1.072	0.999, 1.150	0.0543
Salty	117,396	1,814	1.198	1.108, 1.295	< .0001
Meal preferences					
Vegetables	91,719	1,403	1		
Mixed	345,476	4,591	0.959	0.903, 1.018	0.1714
Meat	47,140	634	1.001	0.911, 1.099	0.9870
Meat consumption frequency (per week)					
≤1 time	105,562	1,819	1		
2–3 times	241,228	3,137	0.982	0.926, 1.040	0.5304
≥4 times	137,545	1,672	0.995	0.931, 1.064	0.8856
Alcohol consumption (g/day)					
0	159,560	2,458	1		
1–14.9	121,400	1,453	1.106	1.036, 1.181	0.0026
15–24.9	72,951	779	1.080	0.995, 1.172	0.0654
25 or more	130,424	1,938	1.237	1.165, 1.314	< .0001
Smoking amount					
Never	153,769	2,068	1		
Ex-smoker	60,626	906	1.158	1.071, 1.252	0.0002
0.5 pack currently	49,329	873	1.257	1.161, 1.36	< .0001
0.5–1 pack currently	141,960	1,850	1.349	1.266, 1.437	< .0001
1 pack currently	78,651	931	1.389	1.284, 1.502	< .0001
Physical activity					
None	286,131	4,361	1		
Low	55,855	546	0.902	0.824, 0.986	0.0237
Moderate to High	142,349	1,721	0.941	0.890, 0.995	0.0342

HR, hazard ratio; CI, confidence interval; BMI, body mass index; SD, standard deviation.

**Table 4:** Risk factor distributions between gastric cancer patients and gastric cancer-free patients (women), and age-adjusted univariable model in the validation cohort.

	Frequency		Age-adjusted univariable model		
	No. of participants at baseline (N = 466,013)	No. of event (N = 2,920)	HR	95% CI	*p*
Age (/year)					
Mean (SD)	51.08 (12.05)				
(Age-Mean_age_)					
(Age-Mean_age_)^2^					
BMI (kg/m^2^)					
<18.5	17,112	135	1.117	0.934, 1.335	0.2267
18.5–22.9	186,486	1,136	1		
23.0–24.9	112,438	685	0.932	0.848, 1.025	0.1447
≥25	149,977	964	0.920	0.845, 1.003	0.0580
Family history of cancer					
No	404,012	2,538	1		
Yes	62,001	382	1.238	1.111, 1.380	0.0001
Meal regularity					
Regular	269,839	1,817	1		
Intermediate	142,957	847	1.017	0.937, 1.103	0.6954
Irregular	53,217	256	0.944	0.828, 1.077	0.3922
Salt preference					
Not salty	70,418	432	1		
Intermediate	308,774	1,896	1.118	1.007, 1.242	0.0366
Salty	86,821	592	1.166	1.030, 1.320	0.0153
Meal preferences					
Vegetables	152,859	1,095	1		
Mixed	292,475	1,734	0.960	0.89, 1.036	0.2970
Meat	20,679	91	0.781	0.63, 0.967	0.0234
Meat consumption frequency (per week)					
≤1 time	167,033	1,277	1		
2–3 times	222,641	1,265	0.937	0.866, 1.013	0.1023
≥4 times	76,339	378	0.914	0.814, 1.026	0.1286
Alcohol consumption (g/day)					
0	384,388	2,480	1		
1–14.9	66,809	344	0.995	0.888, 1.114	0.9270
15 or more	14,816	96	1.110	0.905, 1.361	0.3162
Smoking amount					
Never	439,667	2,703	1		
smoker	26,346	217	0.988	0.859, 1.136	0.8658
Physical activity					
None	361,973	2,352	1		
Yes	104,040	568	0.944	0.861, 1.035	0.2227

HR, hazard ratio; CI, confidence interval; BMI, body mass index; SD, standard deviation.

### Illustration of predicted risk probability based on various risk profiles

**Fig 3:**
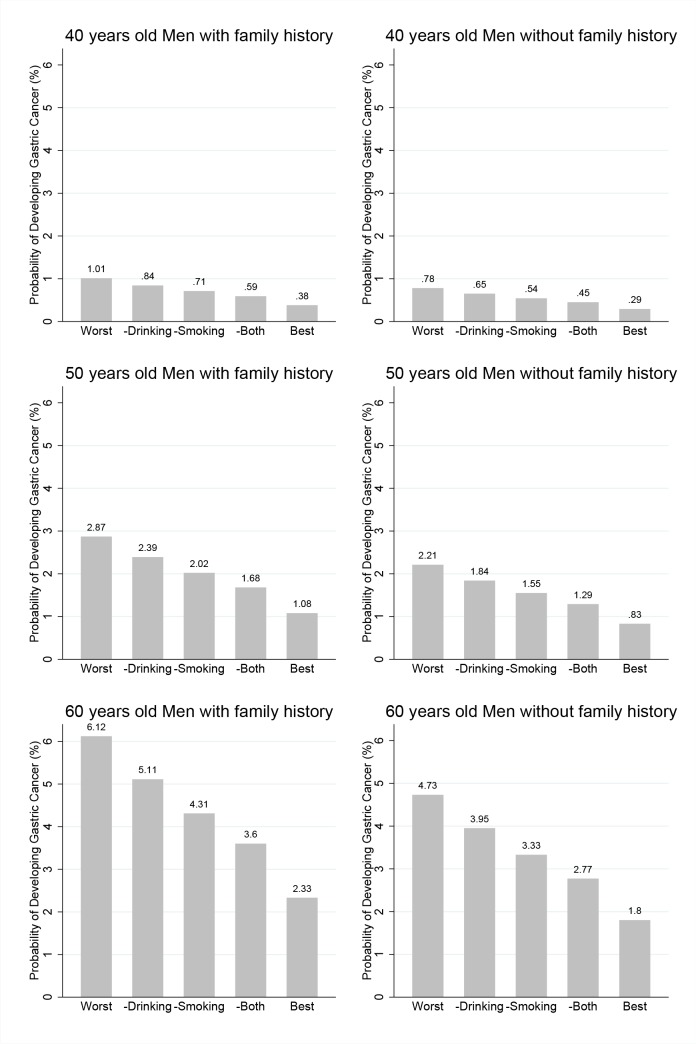
Estimated probabilities of developing gastric cancer for men. Estimated probabilities of developing gastric cancer within 8 years for men for ages 40 (top), 50 (middle) and 60 (bottom). The left and right panels present these estimates of subjects with and without family history of any cancer, respectively. The risk combinations for each category of the X-axis are as follows. Worst corresponds to a BMI < 18.5 kg/m^2^; Meal regularity, Irregular; Salt preference, Salty; Alcohol consumption, ≥ 25 g/day; Smoking, 1 pack currently; and Physical activity, None.–Drinking is the same except that Alcohol consumption is 0, and–Smoking is the same except that the Smoking amount is None.–Both is the same except the Smoking amount is None and the Alcohol consumption is 0. Best corresponds to a BMI ≥25; Meal regularity, Regular; Salt preference, Not salty; Alcohol consumption, 0; Smoking amount, Never; and Physical activity, Moderate to high.

**Fig 4:**
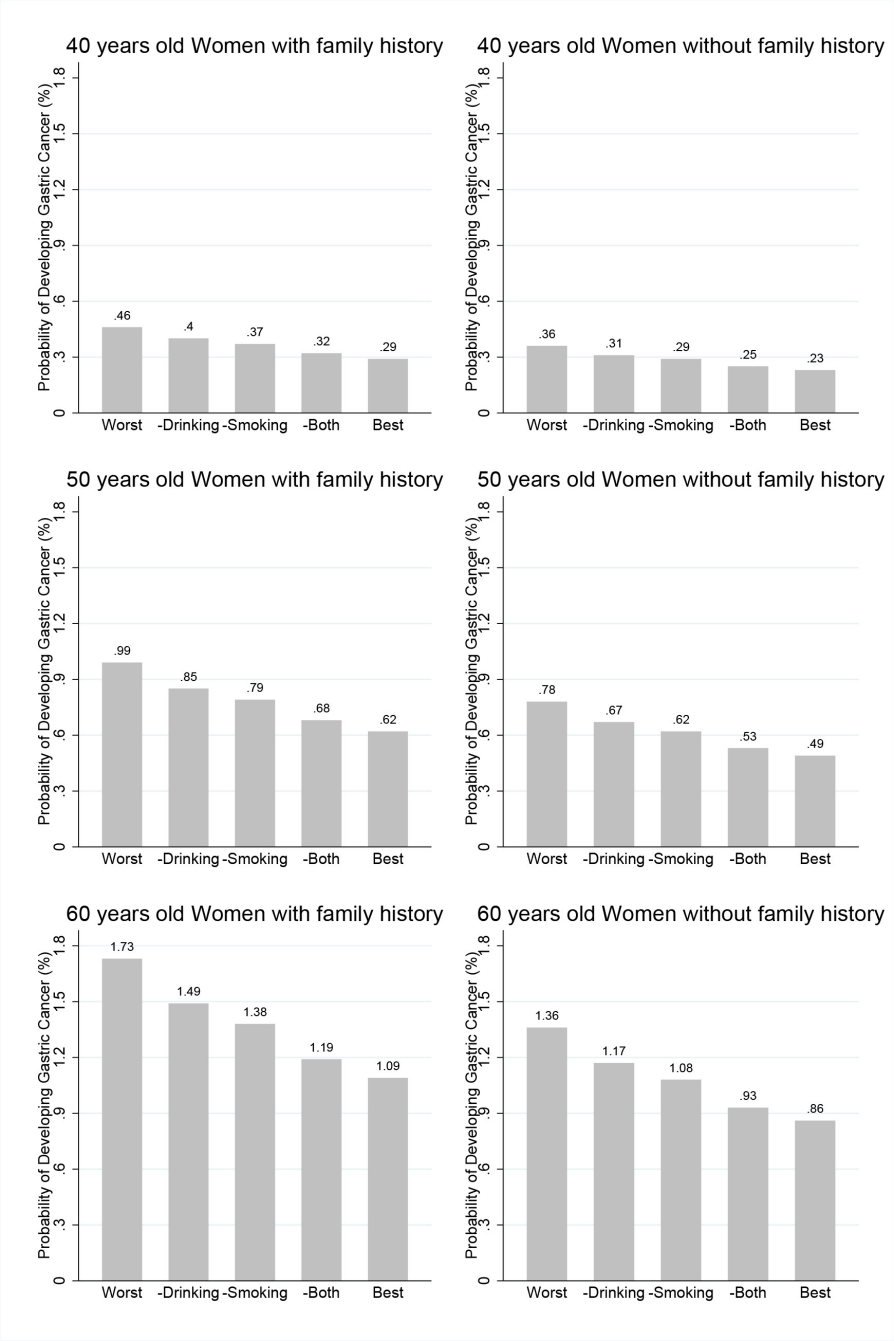
Estimated probabilities of developing gastric cancer for women. Estimated probabilities of developing gastric cancer within 8 years for women for ages 40 (top), 50 (middle) and 60 (bottom). The left and right panels present these estimates of subjects with and without family history of any cancer, respectively. Worst corresponds to a BMI < 18.5 kg/m^2^; Salt preference, Salty; Alcohol consumption, ≥15g/day; and Smoking amount, Smoker.–Smoking is the same except the Smoking amount is Smoker, and–Drinking is the same except the Alcohol consumption is 0. Best corresponds to a BMI ≥25; Salt preference, Not salty; Alcohol consumption, 0; and Smoking amount, 0.

In Figs 3 and 4, the estimated probabilities of developing gastric cancer within 8 years are presented for men (Fig 3) and women (Fig 4) for ages 40 (top row), 50 (middle row) and 60 (bottom row). The left and right panels present these estimates for subjects who have family members with and without any cancer, respectively. For men, the leftmost Fig represents the risk probability for a person with the worst risk combination, that is, a thin person (BMI<18.5 kg/m^2^) who is a heavy smoker and drinker, has irregular meals, prefers salty food and does not exercise. The risk probabilities of a man with same risk combinations except alcohol consumption or except smoking are presented in the next two plots. Then, the plot for a man with same risk combinations but without alcohol consumption or smoking is presented. Finally, the plot for a man without any risk factors is presented. This last Fig presents the risk probability of this person under the best risk combinations. Similarly, for women, the leftmost Fig is presents for women with the worst risk factors, and their risk probabilities when only smoking, only alcohol consumption, or both are removed from the risk factors; these risk probabilities are presented in turn. Finally, the last Fig is represents for women without any risk factors.

For example, we can consider a man who is 50 years old with a family member with any type of cancer. The probability of developing gastric cancer within 8 years can be as high as 2.87% under the worst risk combinations. If a man who has the same risk combinations does not drink alcohol, the risk is 2.39%; if both smoking and alcohol consumption are removed from the risk combinations, the risk decreases to 1.68%. Finally, a lowest possible risk value of 1.08% is present in a man with no risk factors, which is less than half the value for the worst combinations.

## Discussion

Many epidemiological studies have evaluated the risk factors of gastric cancer incidence. However, there have been only a few studies that have developed prediction models for gastric cancer incidence. In the present study, gender specific predictive models for gastric cancer incidence were developed and validated based on a large population-based cohort. Low weight, a family history of cancer, irregular meals, preference for salty food, alcohol consumption, smoking, and a lack of physical activity were related with developing gastric cancer for men; low weight, a family history of cancer, preference for salty food, alcohol consumption, and smoking were associated with developing gastric cancer for women.

Risk factors for gastric cancer incidence have been revealed in previous studies. The most typical risk factor is *H*. *pylori* infection; this has been classified as carcinogenic to humans since 1994 [22]. Smoking has also been acknowledged as one of the causes of gastric cancer by the International Agency for Research on Cancer since 2004 [23]. Probable risk factors include preferences for salt, salty and smoked foods, and heavy alcohol consumption [24,25]. Conversely, green-yellow vegetables, allium vegetables and fruits, and citrus fruits are probable protective factors [24,26]. Moreover, red and processed meats, haem iron, and obesity (for cardia) are possible risk factors, whereas estrogen is a possible protective factor [27–30]. Family history is also associated with gastric cancer incidence, with an odds ratio ranging from 2 to 10 [31,32]. Among these known risk factors, we included 10 risk factors that could easily be collected by a simple physical examination or questionnaire. Data for *H*. *pylori* infection and specific foods such as allium vegetables could not be collected because an invasive procedure for H. pylori and a trained interviewer for specific foods were not included in this study.

In this study, we observed that BMI was a protective factor in the male population. Previously, some studies showed an increased risk of gastroesophageal cancer incidence in overweight subjects, and other studies reported no significant relationship between being overweight and overall gastric cancer incidence [33–35]. However, a recent meta-analysis revealed that being overweight is a protective factor for non-cardia cancer, and a similar pattern of hazard ratios was observed in a large-scale cohort study [36, 37]. Because the majority of gastric cancer cases were located in the distal part of the stomach in Korea and we had a huge sample size, BMI likely had a statistical significance as a protective factor in this study.

Previously, a Korean prediction model for gastric cancer was reported in 2009 [13]. In this study, only three hospitals participated, and less than 200 cases were included as the case and control groups, respectively. However, our prediction models were derived from a nationwide database with more than two million participants and government employees, teachers, company employees and their dependents, which can represent the entire Korean population because these occupational characteristics comprise a large proportion of the entire Korean population. The other advantage of this study is that an external validation using a large sized population was performed, whereas the previous model was not validated in independent data. Moreover, 16 factors were included in the previous model which is somewhat complicated to apply nationwide; however, we included only 8 factors for men and 6 factors for women to predict gastric cancer. Using this model, we can simply predict the risk of gastric cancer development, and high risk groups can easily be identified.

This model can be used when a primary physician counsels healthy individuals after a routine check-up. A primary physician can give a warning for risk factors of gastric cancer after a simple history taking, and each examinee could receive the warning more seriously with an exact probability of gastric cancer. Moreover, if this prediction model is known to the general Korean population, people with high risk factors could be motivated to perform routine check-ups. Additionally, more frequent and intensive screening programs can be implemented to the high risk populations. These active screening could allow gastric cancer to be detected at an earlier stage and might finally result in lowering gastric cancer related mortality.

This study had a few limitations. *H*. *pylori* information was not available because all the data were collected through routine physical examinations. In many countries, H. pylori examination is not included in gastric cancer screening because it requires invasive procedures such as blood sampling or endoscopic examinations, and costs a great deal. Without the invasive procedure of *H*. *pylori* examination, we can predict the risk of gastric cancer development using this model, and this prediction model can be applied to a larger population.

Second, socioeconomic status, educational attainment, and specific foods such as fish, soybeans, allium vegetables, and tea were not considered in this study [38–42]. For these data, a trained interviewer and the interviewee’s effort such as a diet diary are required. These complicated data can be helpful to develop a more delicate model; however, it can also be difficult to generalize.

Third, this study is not free from recall bias because of using a questionnaire for dietary patterns. Additionally, the categories of meal regularity and salt or meal preference were very simple and subjective. However, we suggest these simplified subjective categories of dietary patterns can provide a widely available model for the general population.

Fourth, this study did not assess the risk probability of developing gastric cancer according to tumor location. In some previous studies, smoking and a high BMI tended to increase the risk of cardia cancer, and salty food was positively associated with noncardia (distal) gastric cancer [43–45]. Possibly because of the small number of incidences of cardia cancer, no meaningful distinction between risk factors for cardia and distal cancers was observed in the current study (data not shown). Further study would be worth pursuing.

Fifth, the data provided by the NHIC contained a large amount of missing data, and we imputed these missing values based on the data of the nearest time point. This method may not be optimal; however, we suspected that most of the variables did not change within a short period of time. When we developed a prediction model with complete data as a comparison, only meal regularity for men and BMI and salt preference for women were eliminated in the model with complete data because of the reduced statistical significance resulting from the reduced sample size. Therefore, we concluded that the effect of the missing data on the model development will be minor.

Sixth, this prediction model was validated by a similar Korean population and the prediction of this model may be limited to the Korean population.

In conclusion, we can assess the risk of gastric cancer incidence using age, BMI, a family history of any cancer, meal regularity, salt preference, alcohol consumption, smoking, and physical activity for men, and using age, BMI, a family history of any cancer, salt preference, alcohol consumption, and smoking for women. This simple tool for the general public may be helpful to educate and motivate individuals to participate in screening programs.

## Supporting Information

S1 File Risk factor distributions between gastric cancer patients and gastric cancer-free patients (men), and age-adjusted univariable and multivariable model in the complete developing cohort (Table A). Risk factor distributions between gastric cancer patients and gastric cancer-free patients (women), and age-adjusted univariable and multivariable model in the complete developing cohort (Table B). Risk factor distributions between gastric cancer patients and gastric cancer-free patients (men), and age-adjusted univariable model in the complete validation cohort (Table C).Risk factor distributions between gastric cancer patients and gastric cancer-free patients (women), and age-adjusted univariable model in the complete validation cohort (Table D).
(DOCX)
