# Supplementary material for: Prediction Model for Gastric Cancer Incidence in Korean Population
Source: PLoS One. 2015 Jul 17;10(7):e0132613. doi: 10.1371/journal.pone.0132613 (PMC4506054; doi:10.1371/journal.pone.0132613)
Supplement: S1 File — Risk factor distributions between gastric cancer patients and gastric cancer-free patients (women), and age-adjusted univariable and multivariable model in the complete developing cohort (Table B). Risk factor distributions between gastric cancer patients and gastric cancer-free patients (men), and age-adjusted univariable model in the complete validation cohort (Table C).Risk factor distributions between gastric cancer patients and gastric cancer-free patients (women), and age-adjusted univariable model in the complete validation cohort (Table D). (DOCX) [file pone.0132613.s001.docx]

Table A. Risk factor distributions between gastric cancer patients and gastric cancer-free patients (men), and age-adjusted univariable and multivariable model in the complete developing cohort.

|  | | Frequency | | |  | | Age-adjusted univariable model | |  | Multivariable model | | | | | | |  |
| --- | --- | --- | --- | --- | --- | --- | --- | --- | --- | --- | --- | --- | --- | --- | --- | --- | --- |
|  | No. of participants at baseline (N=823,741) | | | No. of event (N=11,578) | HR | 95 % CI | | *p* | HR | | 95 % CI | | | *p* |  | | |
| Age (year) |  | |  | |  |  | |  |  | | |  |  | | |  |  |
| Mean (SD) | 44.80 (10.37) | |  | |  |  | |  | 1.11 | | | 1.107, 1.114 | < 0.001 | | |  |  |
| (Age-Mean_age_) |  | |  | |  |  | |  | 1.11 | | | 1.107, 1.114 | < 0.001 | | |  |  |
| (Age-Mean_age_)^2^ |  | |  | |  |  | |  | 0.999 | | | 0.998, 0.999 | < 0.001 | | |  |  |
| BMI (kg/m^2^) |  | |  | |  |  | |  |  | | |  |  | | |  |  |
| <18.5 | 20,425 | | 409 | | 1.055 | 0.954, 1.167 | | 0.298 | 1.104 | | | 0.997, 1.222 | 0.056 | | |  |  |
| 18.5-22.9 | 341,907 | | 5,030 | | 1 |  | |  | 1 | | |  |  | | |  |  |
| 23.0-24.9 | 231,929 | | 3,142 | | 0.925 | 0.885, 0.967 | | 0.001 | 0.915 | | | 0.875, 0.957 | 0.001 | | |  |  |
| ≥25 | 229,480 | | 2,997 | | 0.911 | 0.871, 0.953 | | < 0.001 | 0.896 | | | 0.856, 0.938 | < 0.001 | | |  |  |
| Family history of cancer |  | |  | |  |  | |  |  | | |  |  | | |  |  |
| No | 672,323 | | 9,147 | | 1 |  | |  | 1 | | |  |  | | |  |  |
| Yes | 151,418 | | 2,431 | | 1.345 | 1.286, 1.407 | | < 0.001 | 1.322 | | | 1.264, 1.383 | < 0.001 | | |  |  |
| Meal regularity |  | |  | |  |  | |  |  | | |  |  | | |  |  |
| Regular | 478,714 | | 7,226 | | 1 |  | |  |  | | |  |  | | |  |  |
| Intermediate | 273,192 | | 3,479 | | 1.092 | 1.048, 1.138 | | < 0.001 |  | | |  |  | | |  |  |
| Irregular | 71,835 | | 873 | | 1.173 | 1.093, 1.26 | | < 0.001 |  | | |  |  | | |  |  |
| Salt preference |  | |  | |  |  | |  |  | | |  |  | | |  |  |
| Not salty | 133,412 | | 1,788 | | 1 |  | |  | 1 | | |  |  | | |  |  |
| Intermediate | 514,589 | | 6,988 | | 1.058 | 1.004, 1.114 | | 0.034 | 1.001 | | | 0.95, 1.055 | 0.966 | | |  |  |
| Salty | 175,740 | | 2,802 | | 1.221 | 1.151, 1.296 | | < 0.001 | 1.066 | | | 1.003, 1.133 | 0.038 | | |  |  |
| Meal preferences |  | |  | |  |  | |  |  | | |  |  | | |  |  |
| Vegetables | 169,496 | | 2,520 | | 1 |  | |  |  | | |  |  | | |  |  |
| Mixed | 585,419 | | 8,081 | | 0.971 | 0.928, 1.015 | | 0.194 |  | | |  |  | | |  |  |
| Meat | 68,826 | | 977 | | 1.007 | 0.936, 1.085 | | 0.847 |  | | |  |  | | |  |  |
| Meat consumption frequency (per week) |  | |  | |  |  | |  |  | | |  |  | | |  |  |
| ≤1 time | 383,720 | | 5,528 | | 1 |  | |  |  | | |  |  | | |  |  |
| 2-3 times | 397,989 | | 5,255 | | 0.986 | 0.949, 1.024 | | 0.461 |  | | |  |  | | |  |  |
| ≥4 times | 42,032 | | 795 | | 1.023 | 0.949, 1.102 | | 0.559 |  | | |  |  | | |  |  |
| Alcohol consumption (g/day) |  | |  | |  |  | |  |  | | |  |  | | |  |  |
| 0 | 245,472 | | 3,667 | | 1 |  | |  | 1 | | |  |  | | |  |  |
| 1-14.9 | 236,249 | | 2,916 | | 1.061 | 1.011, 1.115 | | 0.017 | 1.018 | | | 0.968, 1.069 | 0.4923 | | |  |  |
| 15-24.9 | 145,217 | | 1,845 | | 1.173 | 1.109, 1.242 | | < 0.001 | 1.094 | | | 1.033, 1.159 | 0.0022 | | |  |  |
| 25 or more | 196,803 | | 3,150 | | 1.368 | 1.303, 1.435 | | < 0.001 | 1.235 | | | 1.175, 1.298 | < 0.001 | | |  |  |
| Smoking amount |  | |  | |  |  | |  |  | | |  |  | | |  |  |
| Never | 233,101 | | 2,911 | | 1 |  | |  | 1 | | |  |  | | |  |  |
| Ex-smoker | 123,543 | | 1,949 | | 1.225 | 1.157, 1.297 | | < 0.001 | 1.176 | | | 1.11, 1.246 | < 0.001 | | |  |  |
| 0.5 pack currently | 75,873 | | 1,177 | | 1.281 | 1.197, 1.371 | | < 0.001 | 1.261 | | | 1.178, 1.351 | < 0.001 | | |  |  |
| 0.5–1 pack currently | 274,414 | | 3,859 | | 1.448 | 1.38, 1.52 | | < 0.001 | 1.357 | | | 1.291, 1.427 | < 0.001 | | |  |  |
| 1 pack currently | 116,810 | | 1,682 | | 1.591 | 1.498, 1.691 | | < 0.001 | 1.439 | | | 1.352, 1.532 | < 0.001 | | |  |  |
| Physical activity |  | |  | |  |  | |  |  | | |  |  | | |  |  |
| None | 400,239 | | 5,767 | | 1 |  | |  | 1 | | |  |  | | |  |  |
| Low | 130,308 | | 1,743 | | 0.993 | 0.941, 1.048 | | 0.804 | 0.998 | | | 0.945, 1.053 | 0.938 | | |  |  |
| Moderate | 237,243 | | 3,034 | | 0.946 | 0.905, 0.989 | | 0.014 | 0.946 | | | 0.905, 0.99 | 0.016 | | |  |  |
| High | 55,951 | | 1,034 | | 1.006 | 0.941, 1.075 | | 0.865 | 0.999 | | | 0.935, 1.068 | 0.981 | | |  |  |

HR, hazard ratio; CI, confidence interval; BMI, body mass index; SD, standard deviation

Table B Risk factor distributions between gastric cancer patients and gastric cancer-free patients (women), and age-adjusted univariable and multivariable model in the complete developing cohort.

|  | | Frequency | | | Age-adjusted univariable model | | |  | Multivariable model | | |
| --- | --- | --- | --- | --- | --- | --- | --- | --- | --- | --- | --- |
|  | No. of participants at baseline (N=369,554) | | | No. of event (N=2,603) | HR | 95 % CI | *p* | HR | | 95 % CI | *p* |
| Age (/year) |  | |  | |  |  |  |  | |  |  |
| Mean (SD) | 48.80 (11.11) | |  | |  |  |  | 1.071 | | 1.065-1.076 | <.0001 |
| (Age-Mean_age_) |  | |  | |  |  |  | 1.071 | | 1.065-1.076 | <.0001 |
| (Age-Mean_age_)^2^ |  | |  | |  |  |  | 0.999 | | 0.999-1 | <.0001 |
| BMI (kg/m^2^) |  | |  | |  |  |  |  | |  |  |
| <18.5 | 14976 | | 111 | | 1.153 | 0.947, 1.403 | 0.155 |  | |  |  |
| 18.5-22.9 | 160708 | | 1,004 | | 1 |  |  |  | |  |  |
| 23.0-24.9 | 88431 | | 655 | | 1.018 | 0.922, 1.123 | 0.726 |  | |  |  |
| ≥25 | 105439 | | 833 | | 0.967 | 0.882, 1.061 | 0.478 |  | |  |  |
| Family history of cancer |  | |  | |  |  |  |  | |  |  |
| No | 298646 | | 2,055 | | 1 |  |  | 1 | |  |  |
| Yes | 70908 | | 548 | | 1.305 | 1.187, 1.434 | <.0001 | 1.297 | | 1.18-1.426 | <.0001 |
| Meal regularity |  | |  | |  |  |  |  | |  |  |
| Regular | 213083 | | 1,533 | | 1 |  |  |  | |  |  |
| Intermediate | 112520 | | 765 | | 0.996 | 0.913, 1.086 | 0.922 |  | |  |  |
| Irregular | 43951 | | 305 | | 1.079 | 0.954, 1.22 | 0.226 |  | |  |  |
| Salt preference |  | |  | |  |  |  |  | |  |  |
| Not salty | 62110 | | 445 | | 1 |  |  |  | |  |  |
| Intermediate | 244031 | | 1,646 | | 1.018 | 0.916, 1.13 | 0.743 |  | |  |  |
| Salty | 63413 | | 512 | | 1.101 | 0.97, 1.251 | 0.136 |  | |  |  |
| Meal preferences |  | |  | |  |  |  |  | |  |  |
| Vegetables | 130414 | | 999 | | 1 |  |  |  | |  |  |
| Mixed | 222659 | | 1,489 | | 1.03 | 0.95, 1.116 | 0.477 |  | |  |  |
| Meat | 16481 | | 115 | | 1.106 | 0.912, 1.342 | 0.307 |  | |  |  |
| Meat consumption frequency (per week) |  | |  | |  |  |  |  | |  |  |
| ≤1 time | 217823 | | 1,587 | | 1 |  |  |  | |  |  |
| 2-3 times | 129619 | | 860 | | 0.986 | 0.907, 1.071 | 0.736 |  | |  |  |
| ≥4 times | 22112 | | 156 | | 0.933 | 0.791, 1.1 | 0.407 |  | |  |  |
| Alcohol consumption (g/day) |  | |  | |  |  |  |  | |  |  |
| 0 | 313999 | | 2,251 | | 1 |  |  | 1 | |  |  |
| 1-14.9 | 46543 | | 265 | | 0.912 | 0.803, 1.036 | 0.159 | 0.9 | | 0.792-1.023 | 0.108 |
| 15 or more | 9012 | | 87 | | 1.263 | 1.019, 1.564 | 0.033 | 1.21 | | 0.974-1.503 | 0.0853 |
| Smoking amount |  | |  | |  |  |  |  | |  |  |
| Never | 348618 | | 2,348 | | 1 |  |  | 1 | |  |  |
| smoker | 20936 | | 255 | | 1.185 | 1.038, 1.352 | 0.012 | 1.198 | | 1.047-1.37 | 0.009 |
| Physical activity |  | |  | |  |  |  |  | |  |  |
| None | 278150 | | 1,973 | | 1 |  |  |  | |  |  |
| Yes | 91404 | | 630 | | 0.973 | 0.889, 1.064 | 0.5486 |  | |  |  |

HR, hazard ratio; CI, confidence interval; BMI, body mass index; SD, standard deviation

Table C. Risk factor distributions between gastric cancer patients and gastric cancer-free patients (men), and age-adjusted univariable model in the complete validation cohort

|  | | Frequency | | | Age-adjusted univariable model | | |
| --- | --- | --- | --- | --- | --- | --- | --- |
|  | No. of participants at baseline (N=543,081) | | No. of event (N=6,702) | HR | | 95 % CI | *p* |
| Age (/year) |  | |  |  | |  |  |
| Mean (SD) | 46.29 (11.58) | |  |  | |  |  |
| (Age-Mean_age_) |  | |  |  | |  |  |
| (Age-Mean_age_)^2^ |  | |  |  | |  |  |
| BMI (kg/m^2^) |  | |  |  | |  |  |
| <18.5 | 15,198 | | 318 | 1.009 | | 0.898, 1.134 | 0.88 |
| 18.5-22.9 | 210,755 | | 2,903 | 1 | |  |  |
| 23.0-24.9 | 148,835 | | 1,700 | 0.931 | | 0.876, 0.988 | 0.019 |
| ≥25 | 168,293 | | 1,781 | 0.924 | | 0.87, 0.981 | 0.009 |
| Family history of cancer |  | |  |  | |  |  |
| No | 470,890 | | 5,772 | 1 | |  |  |
| Yes | 72,191 | | 930 | 1.314 | | 1.225, 1.408 | < 0.001 |
| Meal regularity |  | |  |  | |  |  |
| Regular | 328,501 | | 4,339 | 1 | |  |  |
| Intermediate | 171,125 | | 1,930 | 1.129 | | 1.069, 1.192 | < 0.001 |
| Irregular | 43,455 | | 433 | 1.151 | | 1.042, 1.271 | 0.006 |
| Salt preference |  | |  |  | |  |  |
| Not salty | 87,561 | | 1,026 | 1 | |  |  |
| Intermediate | 334,558 | | 3,942 | 1.072 | | 1.001, 1.148 | 0.048 |
| Salty | 120,962 | | 1,734 | 1.224 | | 1.133, 1.322 | < 0.001 |
| Meal preferences |  | |  |  | |  |  |
| Vegetables | 107,024 | | 1,383 | 1 | |  |  |
| Mixed | 390,360 | | 4,747 | 1.024 | | 0.965, 1.088 | 0.434 |
| Meat | 45,697 | | 572 | 1.061 | | 0.962, 1.17 | 0.234 |
| Meat consumption frequency (per week) |  | |  |  | |  |  |
| ≤1 time | 97,639 | | 1,529 | 1 | |  |  |
| 2-3 times | 278,433 | | 3,264 | 0.986 | | 0.927, 1.048 | 0.65 |
| ≥4 times | 167,009 | | 1,909 | 1.025 | | 0.957, 1.097 | 0.48 |
| Alcohol consumption (g/day) |  | |  |  | |  |  |
| 0 | 171,091 | | 2,323 | 1 | |  |  |
| 1-14.9 | 146,446 | | 1,615 | 1.127 | | 1.057, 1.202 | < 0.001 |
| 15-24.9 | 89,961 | | 929 | 1.147 | | 1.062, 1.24 | < 0.001 |
| 25 or more | 135,583 | | 1,835 | 1.28 | | 1.204, 1.362 | < 0.001 |
| Smoking amount |  | |  |  | |  |  |
| Never | 173,229 | | 2,015 | 1 | |  |  |
| Ex-smoker | 72,543 | | 963 | 1.179 | | 1.092, 1.274 | < 0.001 |
| 0.5 pack currently | 51,699 | | 764 | 1.2 | | 1.104, 1.304 | < 0.001 |
| 0.5–1 pack currently | 160,924 | | 1,951 | 1.393 | | 1.308, 1.483 | < 0.001 |
| 1 pack currently | 84,686 | | 1,009 | 1.531 | | 1.418, 1.653 | < 0.001 |
| Physical activity |  | |  |  | |  |  |
| None | 285,616 | | 3,915 | 1 | |  |  |
| Low | 69,674 | | 710 | 0.96 | | 0.885, 1.04 | 0.318 |
| Moderate | 153,291 | | 1,426 | 0.886 | | 0.833, 0.942 | 0.001 |
| High | 34,500 | | 651 | 1.127 | | 1.038, 1.225 | 0.005 |

HR, hazard ratio; CI, confidence interval; BMI, body mass index; SD, standard deviation

Table D. Risk factor distributions between gastric cancer patients and gastric cancer-free patients (women), and age-adjusted univariable model in the complete validation cohort

|  | Frequency | | | | Age-adjusted univariate model | | |
| --- | --- | --- | --- | --- | --- | --- | --- |
|  | | No. of participants at baseline (N=391,320) | No. of event (N=2,338) | HR | | 95 % CI | *p* |
| Age (/year) | |  |  |  | |  |  |
| Mean (SD) | | 50.43 (11.94) |  |  | |  |  |
| (Age-Mean_age_) | |  |  |  | |  |  |
| (Age-Mean_age_)^2^ | |  |  |  | |  |  |
| BMI (kg/m^2^) | |  |  |  | |  |  |
| <18.5 | | 15,208 | 112 | 1.178 | | 0.967, 1.434 | 0.104 |
| 18.5-22.9 | | 164,046 | 891 | 1 | |  |  |
| 23.0-24.9 | | 93,386 | 553 | 0.995 | | 0.895, 1.106 | 0.922 |
| ≥25 | | 118,680 | 782 | 1.02 | | 0.926, 1.123 | 0.691 |
| Family history of cancer | |  |  |  | |  |  |
| No | | 330,141 | 1,970 |  | |  |  |
| Yes | | 61,179 | 368 | 1.24 | | 1.109, 1.388 | < 0.001 |
| Meal regularity | |  |  |  | |  |  |
| Regular | | 237,609 | 1,496 | 1 | |  |  |
| Intermediate | | 112,874 | 641 | 0.988 | | 0.9, 1.084 | 0.793 |
| Irregular | | 40,837 | 201 | 0.963 | | 0.83, 1.116 | 0.613 |
| Salt preference | |  |  |  | |  |  |
| Not salty | | 62,029 | 366 | 1 | |  |  |
| Intermediate | | 258,946 | 1,521 | 1.1 | | 0.981, 1.233 | 0.104 |
| Salty | | 70,345 | 451 | 1.113 | | 0.969, 1.277 | 0.129 |
| Meal preferences | |  |  |  | |  |  |
| Vegetables | | 129,479 | 890 | 1 | |  |  |
| Mixed | | 244,447 | 1,359 | 0.935 | | 0.859, 1.018 | 0.123 |
| Meat | | 17,394 | 89 | 0.939 | | 0.754, 1.167 | 0.569 |
| Meat consumption frequency (per week) | |  |  |  | |  |  |
| ≤1 time | | 131,679 | 963 | 1 | |  |  |
| 2-3 times | | 190,962 | 1,040 | 0.944 | | 0.864, 1.032 | 0.205 |
| ≥4 times | | 68,679 | 335 | 0.949 | | 0.837, 1.077 | 0.419 |
| Alcohol consumption (g/day) | |  |  |  | |  |  |
| 0 | | 326,609 | 2,017 | 1 | |  |  |
| 1-14.9 | | 53,936 | 252 | 0.925 | | 0.811, 1.055 | 0.245 |
| 15 or more | | 10,775 | 69 | 1.088 | | 0.856, 1.384 | 0.489 |
| Smoking amount | |  |  |  | |  |  |
| Never | | 371,190 | 2,171 | 1 | |  |  |
| smoker | | 20,130 | 167 | 0.997 | | 0.851, 1.169 | 0.973 |
| Physical activity | |  |  |  | |  |  |
| None | | 300,008 | 1,809 | 1 | |  |  |
| Yes | | 91,312 | 529 | 1.057 | | 0.959, 1.165 | 0.261 |

HR, hazard ratio; CI, confidence interval; BMI, body mass index; SD, standard deviation
